# Chemical or Biological Terrorist Attacks: An Analysis of the Preparedness of Hospitals for Managing Victims Affected by Chemical or Biological Weapons of Mass Destruction

**DOI:** 10.3390/ijerph2006030008

**Published:** 2006-03-31

**Authors:** Russell L. Bennett

**Affiliations:** 1 Department of Health Policy & Management, School of Public Health, College of Public Service, Jackson State University, Jackson Medical Mall, 350 W. Woodrow Wilson Ave., Suite 2301-A, Jackson, Mississippi, USA

**Keywords:** Hospital, Terrorism, Preparedness, Weapons of Mass Destruction

## Abstract

The possibility of a terrorist attack employing the use of chemical or biological weapons of mass destruction (WMD) on American soil is no longer an empty threat, it has become a reality. A WMD is defined as any weapon with the capacity to inflict death and destruction on such a massive scale that its very presence in the hands of hostile forces is a grievous threat. Events of the past few years including the bombing of the World Trade Center in 1993, the Murrah Federal Building in Oklahoma City in 1995 and the use of planes as guided missiles directed into the Pentagon and New York’s Twin Towers in 2001 (9/11) and the tragic incidents involving twenty-three people who were infected and five who died as a result of contact with anthrax-laced mail in the Fall of 2001, have well established that the United States can be attacked by both domestic and international terrorists without warning or provocation. In light of these actions, hospitals have been working vigorously to ensure that they would be “ready” in the event of another terrorist attack to provide appropriate medical care to victims. However, according to a recent United States General Accounting Office (GAO) nationwide survey, our nation’s hospitals still are not prepared to manage mass causalities resulting from chemical or biological WMD. Therefore, there is a clear need for information about current hospital preparedness in order to provide a foundation for systematic planning and broader discussions about relative cost, probable effectiveness, environmental impact and overall societal priorities. Hence, the aim of this research was to examine the current preparedness of hospitals in the State of Mississippi to manage victims of terrorist attacks involving chemical or biological WMD. All acute care hospitals in the State were selected for inclusion in this study. Both quantitative and qualitative methods were utilized for data collection and analysis. Six hypotheses were tested. Using a questionnaire survey, the availability of functional preparedness plans, specific preparedness education/training, decontamination facilities, surge capacity, pharmaceutical supplies, and laboratory diagnostic capabilities of hospitals were examined. The findings revealed that a majority (89.2%) of hospitals in the State of Mississippi have documented preparedness plans, provided specific preparedness education/training (89.2%), have dedicated facilities for decontamination (75.7%), and pharmaceutical plans and supplies (56.8%) for the treatment of victims in the event of a disaster involving chemical or biological WMD. However, over half (59.5%) of the hospitals could not increase surge capacity (supplies, equipment, staff, patient beds, etc.) and lack appropriate laboratory diagnostic services (91.9%) capable of analyzing and identifying WMD. In general, hospitals in the State of Mississippi, like a number of hospitals throughout the United States, are still not adequately prepared to manage victims of terrorist attacks involving chemical or biological WMD which consequently may result in the loss of hundreds or even thousands of lives. Therefore, hospitals continue to require substantial resources at the local, State, and national levels in order to be *“truly”* prepared.

## Introduction

The possibility of a terrorist attack employing the use of weapons of mass destruction (WMD) on American soil is no longer an empty threat, it has become a reality. A WMD can be defined as any weapon with the capacity to inflict serious injury and/or death and destruction on such a massive scale that its very presence in the hands of hostile forces is a grievous threat. Events of the past few years including the bombing of the World Trade Center in 1993 and the Murrah Federal Building in Oklahoma City in 1995, the use of planes as guided missiles directed into the World Trade Center and Pentagon in 2001 (9/11) and the tragic incidents following this same year involving twenty-three people who were infected and five who died as a result of contact with anthrax-laced mail, have well established that the United States (U.S.) is not immune to attacks by both domestic and international terrorists without warning or provocation. In light of these incidents and the continued threat of future attacks, hospitals have been working vigorously to ensure that they would be prepared to adequately care for victims in the event of another terrorist attack. However, according to a recent United States General Accounting Office (GAO) nationwide survey of hospital preparedness, our nation’s hospitals still are not prepared to manage mass causalities resulting from any type weapons of mass destruction [1].

In the 2005 State of The Union Address, President Bush stated that “the U.S. and its allies are still the targets of terrorist attacks and our lives and the lives of our friends (allied nations) continue to be threatened” [2]. Additionally, the President stated that although the United States has taken the lead in disarming and decreasing terrorists’ capacity, a number of “terrorist cells” still exist. In a June 2004 news conference entitled “New Terrorist Threats,” Attorney General John Ashcroft announced that “terrorists are planning another attack on U. S. soil and it could happen at any time [3].” Ashcroft and Federal Bureau of Investigation (FBI) Director Robert Mueller revealed that al Qaeda believes it is almost ninety percent complete with plans for another large scale attack on the U.S. Ashcroft and Mueller also believe that the recent railway bombing in Madrid has strengthened the resolve of some terrorist groups, motivating them to attack the U.S. again. In addition, both agree that although a number of planned major events occur often all over the U.S. that could be desirable terrorist targets, with the arrest of a possible terrorist in Ohio who planned to bomb a shopping mall, the next terrorist attack may not necessarily be during a major event. Instead, they suggest that terrorists may target so called “soft targets” such as malls, supermarkets or even apartment buildings for their next attack.

According to Central Intelligence Agency (CIA) Director Porter Gross, terrorist groups associated with al Qaeda are at the top on the list of terrorist threats to the U.S. [4]. In a recent statement before the Senate Intelligence Committee, Gross argued that despite gains made against al Qaeda, terrorist groups are intent on finding ways to circumvent the U.S.’s security enhancements to attack again on American soil. Gross warned the Senate Intelligence Committee that “it may be only a matter of time before al Qaeda or other terrorist groups attempt to use chemical, biological, radiological or nuclear weapons here in the U.S.”

Mike Nartker, a Global Security Newswire writer, recently reported that according to a CIA Think Tank report prepared by the National Intelligence Council (January 2005), a terrorist group is “likely” to conduct an attack in the U.S. using biological weapons by the year 2020 [5]. The report pointed out that over the next fifteen years, successes in the global war on terrorism and advances in information technology are likely to result in an increasingly decentralized terrorist threat, consisting of an eclectic array of terrorist groups, cells and individuals. The report also suggested that acts of biological terrorism would be particularly suited for these smaller and better informed terrorists and that their laboratories need be only the size of a household kitchen and their weapons built smaller than a toaster. The National Intelligence Council Report echoed the warnings of Defense Secretary Donald Rumsfeld who announced in a January 2005 House Armed Services Committee meeting that “terrorists are regrouping for another attack in the U.S.” and we are preparing to deal with this threat [6]”.

As concluded by Rumsfeld, the U.S. has taken seriously the need to prepare for terrorist attacks involving weapons of mass destructions (WMD). For example, Presidential Directive 39, created in 1995, triggered a number of actions among many national agencies to develop strategies that would better position the U.S. for being better prepared to manage incidents involving chemical or biological warfare agents [7]. In 1996, Congress enacted the Defense Against Weapons of Mass Destruction Act, requiring each State to develop a Domestic Preparedness Program and other efforts to improve the capabilities of local emergency response agencies. This program recognized that hospitals and other public health infrastructure are an essential component of the preparedness for terrorist attacks and other hazards both natural and manmade. But despite these general preparedness efforts within the health care industry, hospitals typically are a weak link in the preparedness infrastructure, particularly for those incidents involving chemically or biologically contaminated patients [8].

Since the mid 1990s, approximately 120 of the largest United States cities have received tens of millions of dollars annually in federal funding for training, practice exercises, and equipment to respond to chemical or biological attacks. The Department of Health and Human Services (HHS) has established the Office of Public Health Preparedness to address the preparedness needs of Public Health agencies. In January 2002, President Bush approved $1 billion for preparedness to be administered by the Department of Health and Human Services, to help States prepare their public health infrastructures. In June 2002, an additional $4.6 billion was provided for stockpiling medicines, vaccines and enhancing inspections of the U.S. water-system security. In November 2002, Congress approved the development of the Department of Homeland Security, designed to consolidate several U.S. departments responsible for defenses against terrorist attacks and better coordinate counterterrorism intelligence [9]. In December 2002, the Bush administration announced plans to start inoculating some 10 million healthcare, emergency service, law enforcement, and military personnel against smallpox before making the smallpox vaccine available to the general public on a voluntary basis in 2004. In President Bush’s January 2003 State of the Union address, he proposed spending an additional $6 billion for research and production of vaccines and other treatments against chemical and biological weapons of mass destruction (CBWMD) [10]. Also in January 2003, the Bush administration created a surveillance system designed to detect the release of such deadly germs as anthrax and smallpox within 24 hours by adapting many of the Environmental Protection Agency’s 3,000 air-quality monitor stations nationwide. The Bioterrorism Hospital Preparedness Program, administrated by HHS’s Health Resources and Services Administration (HRSA), provided funding in the fiscal year 2002 in the amount of approximately $125 million through cooperative agreements to States and eligible municipalities to enhance the capacity of hospitals and healthcare entities to manage victims exposed to CBWMD.

Additionally, agencies such as the American Hospital Association, American College of Emergency Physicians, Centers for Disease Control and Prevention, Center for Civil Biodefense Strategies, Joint Commission on Accreditation of Healthcare Organizations and others have established guidelines for the preparedness of hospitals for managing patients exposed to CBWMD [11]. These efforts have assisted in the improvements of U.S. hospitals for managing victims of terrorist attacks involving the use of CBWMD.

Despite these aforementioned policy changes and progress in the preparedness of hospitals for managing victims exposed to CBWMD, scholars contend that a large proportion of hospitals still remain poorly prepared [12]. Donald Henderson suggest that it is common for hospitals to not be fully prepared to respond to mass casualty disasters of any kind, neither in their capacity to care for large numbers of victims or in their ability to provide care in coordination with regional or federal incident command structures. Several surveys of hospital emergency departments have also found broadly prevalent deficits in knowledge, plans, and resources for responding to hazardous materials incidents [13]. James Burgess agrees with Henderson and contends that even relatively small-scale hazardous materials incidents have overwhelmed the response capacities at many hospitals and often resulting in secondary exposure among emergency department staff n ecessitating hospital evacuations [14].

In support of Burgess’ contentions, a national survey of hospitals by the General Accounting Office (GAO) reported recently to a Congressional Committee that hospitals and their emergency departments would require far greater resources than those needed for everyday performance to respond to a large-scale chemical or biological weapons attack [15]. The GAO Report is the result of a review of over 2,000 hospitals in the U.S. to examine the extent to which hospitals are prepared to manage victims of an attack involving WMD. Other findings included in the report were that specific preparedness equipment, supplies, and facilities needed during and after an attack involving CBWMD could vary depending upon what type of terrorist attack. The report also suggested that the demand for healthcare services could quickly outweigh the ability and capacity of hospitals to effectively and efficiently respond to the needs of attack victims.

Several practice scenarios have also been presented to assess the preparedness and response of healthcare systems in the event of a terrorist attack. One such scenario, “TOPOFF 2000”, simulated a terrorist attack using pneumonic plague released at a public event in a single location in one city [16]. In this exercise, officials found that by the third day following the covert release of the toxin, 500 persons with symptoms had been reported and antibiotic shortages were beginning to occur. There was also a shortage of mechanical ventilators needed for respiratory support. By the end of this day, nearly 800 cases had been identified and over 100 persons had died. In each of the succeeding two days, the situation worsened and shortage of medical care within the city was described as critical, with insufficient hospital staff, beds, ventilators, and medications. At the conclusion of the exercise one week after the attack, an estimated 3,700 cases of plague had been identified and 2,000 death s reported. These numbers included cases in other U.S. cities and cities abroad. In the early stages of the epidemic, hospitals were seeing two to three times their normal volume of patients and later in the exercise up to ten times the normal volume were arriving at hospitals. Alarmingly, hospitals were not able to effectively isolate patients to prevent the spread of the disease to hospital staff.

Another practice scenario to test the nation’s healthcare systems for managing victims of terrorist attacks was conducted on June 22–23, 2001 by the John Hopkins Center for Civilian Biodefense Strategies [17]. In collaboration with the Center for Strategic and International Studies, Analytic Services Institute for Homeland Security, and Oklahoma National Memorial Institute for the Prevention of Terrorism, the scenario, “Dark Winter,” simulated a covert smallpox attack on the U.S. Dark Winter was constructed to examine the challenges that senior-level policy makers would face if confronted with a biological terrorist threat that initiated outbreaks of a highly contagious disease. One of the many discoveries in this scenario was that U.S. healthcare systems lacked the capacity to deal with mass casualties. Hospital influx systems across the country were flooded with demands for patient care which consisted of clients who had contracted smallpox, those with common illness and feared they had smallpox, and those who were not ill but worried that they might become infected. The challenges of distinguishing the infected or exposed from those who were not, rationing of scarce resources, and the shortages of health care professionals, who were themselves worried about becoming infected or transmitting the disease to their families, imposed a major burden on all hospitals and other healthcare systems in the nation.

Since September 11, 2001, significant resources have been focused on chemical and biological terrorism planning and preparedness. As indicated by several scholars, these resources have dramatically improved the nation’s ability to confront an attack involving smallpox or other insidious chemical or biological agents. However, according to Victoria Elliott, gaps in the preparedness of hospitals to manage victims resulting from chemical or biological exposure in the long-neglected health care sector still exist [18]. It is these gaps that continue to trigger uncomfortable doubts about the health care system’s surge capacity and other preparedness elements should a worst-case scenario occur. In addition, most health care agencies would not have the capacity to provide large-scale vaccinations or care in the event of a major infectious incident. These sentiments are echoed among healthcare providers and health scholars across the country from cities with varying populations and levels of awareness.

U.S. Representative Barney Frank of Massachusetts was recently asked by the Massachusetts Nursing Association if he believed that hospitals were ready for a terrorist attack. He responded by saying that “hospitals are not ready for Saturday night, let alone a terrorist attack [19].” According to Barney, many hospitals are finding it hard just to keep their doors open which unfortunately could not have happen at a more critical time in our nation’s history. He indicated that hospitals now are faced with the need to prepare themselves to be on the receiving end of the fallout from terrorist attacks thereby serving as the core of the community’s plans to receive, identify and treat contaminated victims. Representative Barney argues that this expectation will be difficult to meet given that most hospitals do not have the proper financial resources to meet such need. The Trust for America’s Public Health, a nonpartisan public health watchdog, recently provided a similar report that supports Representative Barney’s contentions. The Trust agreed that U.S. hospitals are ill prepared to manage victims of a terrorist attack utilizing WMD. In December 2004, the Trust reported that the nation’s public health system is still woefully unprepared to handle a biological terrorist attack and that federal policies are still ill-defined and inconsistent [20].

Clearly, there is a need for more information about the current state of hospitals’ preparedness for managing victims of terrorist attack(s) in an effort to provide a broader foundation for systematic planning and discussions about the necessary resources needed to be truly prepared. To provide such information, this research examined six critical elements of preparedness necessary for hospitals to be truly prepared to manage victims of terrorist attacks involving chemical or biological weapons of mass destruction. These critical elements include: documented/functional preparedness plans; specific preparedness education/training; availability of decontamination facilities; surge capacity; pharmaceutical procedures and supplies; and availability of laboratory diagnostic services capable of analyzing and identifying chemical or biological warfare agents.

## Materials and Method

While much has been done in the State of Mississippi as with hospitals throughout the United States to prepare hospitals for managing victims effected by chemical or biological warfare agents, concerns among citizens, healthcare executives and policymakers remains related to the extent and effectiveness of such preparedness. Therefore, this study is important in that it is focused on current issues of the preparedness of hospitals for managing victims affected by chemical or biological weapons of mass destruction. The findings from this study will provide current information that is paramount to citizens, healthcare executives and policymakers for engaging in broader discussions about the preparedness needs of hospitals specifically in the State of Mississippi in order to be *“truly”* prepared for managing victims of a terrorist event.

### Survey Population

All 102 licensed acute care hospitals in the State of Mississippi were selected for inclusion in this study. *Acute care hospital* refers to the hospital category that best describe the type of services provided to the majority of its patient admissions. This reference includes medical/surgical and a number of the long-term care hospitals in the State. This reference is also consistent with the designation of acute care hospital provided by the Mississippi State Department of Health, Health Facilities Licensure and Certification Division. Additionally, this population selection methodology allowed for the inclusion of hospitals that were located throughout the 82 counties in the State of Mississippi.

This population of hospitals also represents seventy-percent of all hospitals in the State. In addition, these hospitals represent diverse perspectives on the issues of the preparedness of hospitals for managing victims of terrorist attacks involving CBWMD. Hence, it may be concluded that accurate generalizations may be made about the preparedness of hospitals in general related to their ability to manage victims of terrorist attacks in the State of Mississippi.

### Instrumentation/Survey

The Mississippi State Department of Health Directory of Mississippi Healthcare Facilities was used to identify all designated acute care hospitals for possible inclusion in this study. A self-administrated questionnaire survey was constructed and tested for validity and reliability and consisted of two sections. Section one was designed to collect demographic information about the participants. The second section focused on specific preparedness elements necessary for managing victims of an attack involving CBWMD. The questionnaire survey with a cover letter and pre-stamped return envelop was mailed to all acute care hospitals in the State. Follow-up calls were made to hospitals who did not respond by the designated questionnaire return date.

The questionnaire survey contained a total of fifty-three questions of which forty-two required dichotomous responses. Section one requested information about the hospitals’ rural or urban designation; number of hospital and emergency room (ER) beds; percent of the time in the previous year all beds were filled; number of employees; whether or not hospitals had received any preparedness funding; and JCAHO status. Section two of the survey questions were developed based on reoccurring preparedness concepts and variables identified in the review of literature. In addition, a number of the survey questions were developed utilizing the Joint Commission on Accreditation of Healthcare Organization’s (JCAHO) Environment of Care Standards and the American Hospital Association’s (AHA) Chemical an d Bioterrorism Preparedness Checklist, as a frame of reference which are among the most commonly used benchmarks for preparedness. Specifically, section two requested preparedness specific responses related to the availability of documented and functional preparedness plans; specific preparedness education/training; decontamination facilities; surge capacity; pharmaceutical procedures and supplies; and laboratory diagnostic capability.

## Data Analysis

Data were analyzed utilizing the Pearson Product Moment Correlation to determine, if any, a relationship existed between the preparedness of hospitals (dependent variable) and the following selected preparedness variables (independent variables): documented/ functional preparedness plans; specific preparedness education/training; availability of decontamination facilities; surge capacity; availability of pharmaceutical procedures and supplies; and laboratory diagnostic capability. Pearson *r* correlation was used to reflect the degree of linear relationships between variables that ranged from +1.00 to – 1.00. The closer the values were to ± 1.00, the greater the associations were between the variables. The critical values table for Pearson correlation coefficient was used at the .05 level of significance to reflect the probability of an alpha error [21]. Additionally, *Guilford’s Guideline for Interpreting the Strength for Values of r* was utilized as a reference point to interpret the strength of an association, if any, between the dependent variable and independent variables [22]. Demographic data were also analyzed to provide a detailed description of the population under investigation. This data include: location of hospitals (rural or urban); number of hospital and emergency room beds; number of annual patient visits; whether the hospitals were part of a larger health system; whether hospitals received any preparedness funding; and the current accreditation status of each hospital. All analyses were conducted using SPSS for Windows.

## Results

### Demographic Analysis

**Figure 1:**
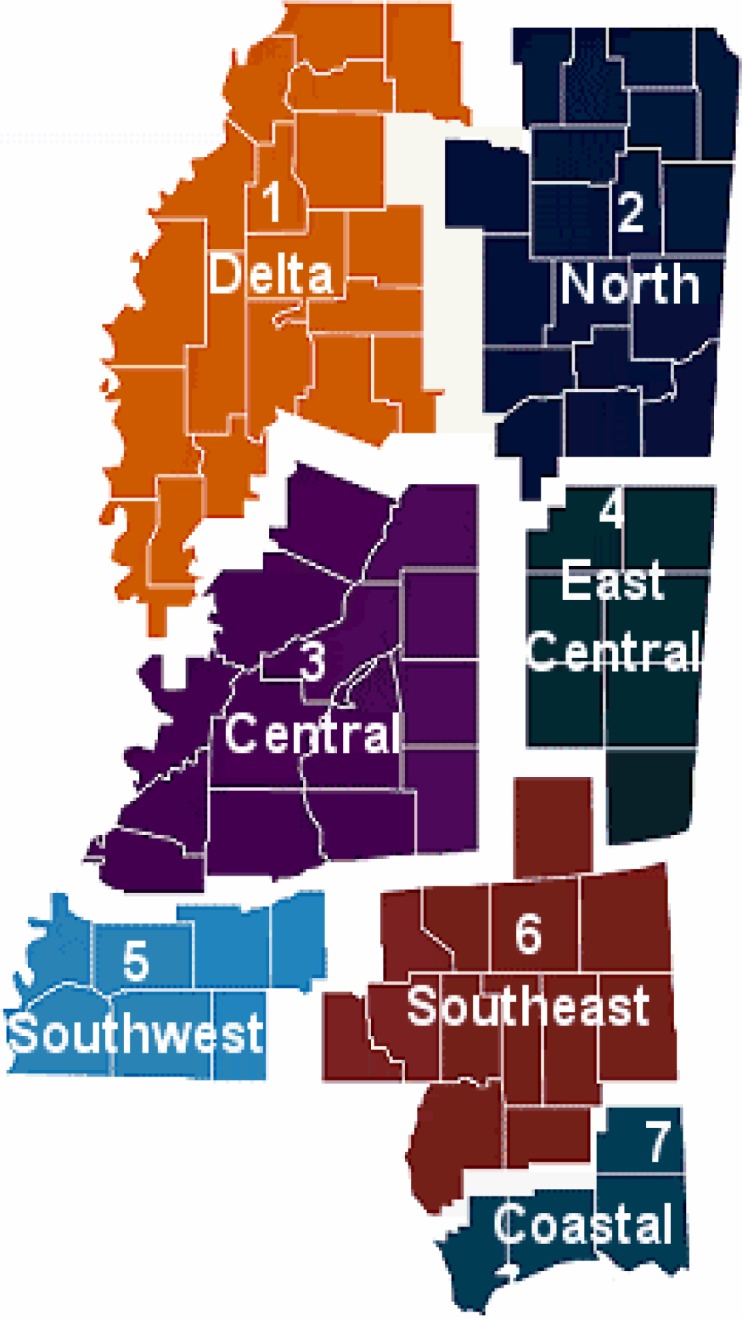
Mississippi Trauma Care System – Trauma Care Region **Source:** Mississippi State Department of Health, Division of Emergency Medical Service (2000).

The State of Mississippi is comprised of 82 counties and 102 acute care hospitals. Figure 1 presents a demographic view of Mississippi, utilizing the Mississippi State Department of Health’s seven Trauma Care Region designations. These designations are commonly used in research efforts and descriptive statistics in the State of Mississippi to provide a framework for discussions about various issues such as the State’s health status, emergency services, hospitals and disaster preparedness efforts. Therefore, these designations were used in this study as reference points for the investigation and description of the preparedness of hospitals for managing victims of a terrorist attack involving chemical or biological weapons of mass destruction.

The majority (27%) of the responses were received from the Central Region of the State, which also has the largest number of hospitals (Table 1). The second largest or 18.9% of the responses was from the Delta Region. The third largest (16.3%) number of responses was from the North Region of Mississippi. The Southwest and Coastal Regions each accounted for 10.8% of the responses. East Central (8.1%) and Southeast (2.7%) were the locations of the least number of responses.

A distinction was made between urban and rural areas for the sake of analysis in that Mississippi has fewer urban areas, in the more common use, than are found in more densely populated States such as Georgia, New York, and the like. The majority of the acute care hospitals (59.5%) were located in urban areas and the remaining 40.5% were located in rural areas of Mississippi (Table 1). Most of the hospitals (48.6%) had between 100 to 300 acute care patient beds, 35.2% having less than 100 and 16.2% with more than 300 hospital beds as shown in Table 1. In addition, the majority (45.9%) of the hospitals had 10 to 20 emergency department beds with 29.8% having less than 10 beds and only 24.3% having more than 20 emergency department beds (Table 1).

Lastly, a majority of the hospitals (67.6%) were not part of a larger health system and had not received any government funding for preparedness (73%) as shown in Table 1. Contrary to these findings, over half (78.4%) of the hospitals were accredited with full standard by the Joint Commission on Accreditation of Healthcare Organizations.

### Documented and Functional Preparedness Plans

The majority of the responding hospitals (89.2%) had documented and functional preparedness plans as shown in Table 2. The questionnaire survey contained eighteen items that measured documented and functional preparedness plans. The critical correlation value for the total sample was .325 (p < 0.05). Therefore, all correlations that were greater than .325 were considered significant at the 0.05 level. All but three measures were identified as significantly correlated to the preparedness of hospitals. However, the majority of the hospitals were not able to identify an alternate point of care individual, augment their security forces, or provide offsite information systems backup.

### Specific Preparedness Education/Training

Specific preparedness education /training was measured by fifteen questionnaire survey items. Ten measures were identified as significantly correlated to the preparedness of hospitals at the .325 (< 0.05) confidence level. However, the respondents reported that they lacked any method for assessing preparedness needs of their staff, providing training while their employees were wearing full personal protective equipment (PPE), and for providing preparedness training for all direct and indirect patient care staff. In addition, the hospitals in this study had no process for providing preparedness training to either full or part-time physician staff.

### Availability of Decontamination Facilities

Well over half (75.7%) of the respondents reported having adequate decontamination facilities for victims exposed to chemical or biological contaminants as indicated in Table 2. Utilizing a critical correlation value of .325 (p < 0.05), all three measures of adequate decontamination facilities were found to be significantly correlated to the preparedness of hospitals for managing victims involving chemical or biological terrorist agents. All of the respondents had one or more dedicated decontamination areas, were able to decontaminate multiple contaminated victims simultaneously, and provided decontamination training to their staff within the past twelve months.

### Availability of Pharmaceutical Procedures and Supplies

The majority (56.8%) of the respondents reported that their hospitals had documented pharmaceutical procedures and appropriate supplies for victims and health care providers in the event of a terrorist attack involving chemical or biological agents. Fourteen items were used to measure the availability of pharmaceutical procedures and appropriate supplies. The critical correlation value of the sample was .325 (< 0.05). The majority of the measures were found to be significantly correlated to the preparedness of hospitals even though a number of the hospitals indicated that they could not provide prophylaxis/medications to the providers’ families in the event of a chemical or biological disaster. These findings are indicated in Table 2.

### Ability to Increase Surge Capacity

Thirteen measures were used to determine if hospitals had an ability to increase their surge capacity in the event of an attack involving CBWMD. The findings from these measures revealed that less than half (40.5%) of the hospitals in this study could increase their surge capacity. In the event of an attack involving chemical or biological agents, hospitals reported that they would not be able to adequately care for the homeless, chronically ill, those with mental disabilities, and those who have cultural/ language needs. In addition, hospitals in this study reported that they would not have rapid access to appropriate respiratory protective equipment such as air purifying masks.

### Diagnostic Laboratory Capability

Only 8.1% of the hospitals in this study had diagnostic laboratory services that were capable of analyzing and identifying chemically or biologically contaminated terrorist agents as indicated in Table 2. Five measures were used to determine how capable hospitals’ diagnostic laboratory services were for being able to adequately analyze and identify chemical or biological terrorist agents. Although the majority of the hospitals reported that they have documented procedures for handing and transporting chemical and biological warfare agent, the majority (91.9%) of them reported that they do not have the appropriate diagnostic laboratory equipment and supplies needed to analyze and identify these agents.

## Discussion

The findings in this research revealed that hospitals generally are not “truly” prepared to manage victims of a terrorist attack involving CBWMD. However, many of the hospitals do have at least some of the critical elements of preparedness. Many of the hospitals in the State of Mississippi do have documented and functional preparedness plans, provide specific preparedness education/training, and have appropriate pharmaceutical procedures and supplies needed in the event of an attack involving chemical or biological terrorist agents. Unfortunately, a number of hospitals reported that they could not increase their surge capacity or have diagnostic laboratory services capable of analyzing and identifying chemical or biological warfare agents.

Although hospitals in urban areas were generally better prepared than rural hospitals in Mississippi, only a few of these hospitals fully met the specific criteria used in this study to measure *“true”* preparedness. More urban than rural hospitals participated in this study which may be due to there being almost twice as many urban hospitals in the State than rural. Only the Southeast region of the State had both urban and rural hospitals. The other six regions had either urban or rural hospitals only. The majority of the hospitals had 100 to 300 inpatient beds and between 10 to 20 emergency department beds.

The majority of hospitals was not part of a larger health system and had not received any federal funding for preparedness efforts. It is conceivable that these factors may indicate that hospitals are constrained by the availability and capacity of existing resources required for the preparedness of hospitals for managing victims exposed to chemical or biological warfare agents. However, the majority of the hospitals were accredited by the Joint Commission on Accreditation of Healthcare Organizations (JCAHO). Hence, it is likely that a number of hospitals in this study, in light of reports that they were not prepared based on the study’s measurement criteria, may be better prepared than indicated. In line with being fully accredited by the JCAHO, these hospitals have achieved a predetermined set of standards for preparedness. These standards indicate a desired level of preparedness for being capable of managing victims of a disaster such as one resulting from a CBWMD.

Though most of the hospitals had preparedness plans, many reported that they had not designated an alternate point of care individual, augmented their security forces and had not secured an offsite information systems backup. These measures negatively impact the preparedness of hospitals for being able to manage victims exposed to a chemical or biological warfare agent [22].

A number of hospitals also provided specific preparedness education/training but lacked an appropriate mechanism for adequately assessing the preparedness education/training needs of some of their staff. Many of the hospitals had no systematic way of determining the preparedness education/training needs of the physicians and indirect patient care staff. The Code of Federal Regulations, 29, 1910.120 require that employers provide appropriate education/training to all levels of staff that have the potential of being exposed to hazardous agents [24]. This would suggest that many of the hospitals in the State must develop better assessment tools that would allow them to assess and provide appropriate education to all levels of their staff in order to be *“truly”* prepared.

The findings in this study also indicate that an ability to increase surge capacity was a major issue for the majority of hospitals in the State of Mississippi. Most of them reported that they were not able to increase their current capacity (staff, supplies, equipment, number of beds, etc.) to care for mass casualties in the event of a chemical or biological disaster. These hospitals also indicated that providing additional care/services for those with physical disorders, children and the elderly would overwhelm their resources. Relatively, although most of the hospitals had appropriate pharmaceutical procedures, in the event of a surge of victims, their supplies (prophylaxis/medications) would be limited.

Although a number of hospitals reported having appropriate diagnostic laboratory procedures for an event involving chemical or biological warfare agent exposure, an alarming number of them (91.9%) reported not having the ability to analyze and/or identify any specific warfare agent(s). Even in light of having appropriate decontamination facilities primarily for chemical exposure, a terrorist deployment of a biological warfare agent may result in mass casualties and fatalities by the time it is diagnosed.

Overall, the findings in this research revealed that acute care hospitals, which represent over seventy-percent of all hospitals in the State of Mississippi, are not “truly” prepared to manage victims of terrorist attacks involving CBWMD. Although these study findings are based entirely on self-administered questionnaire surveys that can carry an inherent risk of reporting bias, the individuals that completed the surveys were professionals who should have been well informed and had ready access to the necessary information to appropriately answer the survey questions.

## Conclusion

Clearly, major efforts have been made to improve the preparedness of hospitals in the State of Mississippi and the nation, but there are still tremendous gaps between these efforts and the preparedness status of hospitals as evident from the findings in this study. Therefore, healthcare executives an d policymaker s should utilize the findings in this study to create a broader forum at the local, State and federal levels for discussions about the critical preparedness issues facing hospitals; engage in efforts to conduct a full and thorough assessment of terrorist threats to determine when and where the next attack will occur; what type of weapon will be used; how will the injuries/illnesses and fatalities be minimized; and what is the relative cost of both preparedness and recovery? In addition, healthcare officials must be encouraged to access and utilize federal funding for preparedness to increase surge capacity and acquire needed supplies and equipment for preparedness. Further, hospital officials must augment their security forces in an effort to provide crowd control and facility command; build local, state and federal alliances in an effort to access crucial resources; expand preparedness education/training and practice to include all level (physicians and indirect patient care providers); and upgrade their diagnostic laboratory capability to analyze and identify chemical or biological warfare agents. Lastly, hospital officials and policymakers should examine the current effectiveness and appropriateness of public policy related to reducing barriers to protected groups such as children, the elderly, mentally challenged, homeless, and those with language/cultural needs in the event of a chemical or biological disaster.

## Figures and Tables

**Table 1:** Hospitals Participating in the Research on the Preparedness of Hospitals for Managing Victims of an Attack Involving Chemical or Biological Weapons of Mass Destruction

Participants	Regions							

	*Delta*	*North*	*Central*	*E. Central*	*S. West*	*S. East*	*Coastal*	*Total*
*Location of Respondents*								

*Rural, *n* (%)^a^	7(18.9)	0	0	3(8.1)	4(10.8)	1(2.7)	0	15(40.5)
**Urban, *n* (%)	0	6(16.2)	10(27)	0	0	2(5.4)	4(10.8)	22(59.5)

*Number of Hospital Beds*								

<100^e^	3(42.8)	2(33.3)	3(30)	0	2(66.9)	2(66.9)	1(25)	13(35.2)^d^
100 – 300^e^	4(77.2)	2(33.3)	4(40)	1(33.1)	4(100)	1(33.1)	2(50)	18(48.6)^d^
>300 ^e^	0	2(33.3)	3(30)	0	0	0	1(25)	6(16.2)^d^

*Number of Emergency Beds*								

<10 ^e^	2(28.6)	1(14.3)	4(89.3)	1(14.3)	1(14.3)	1(14.3)	1(25)	11(29.8)^d^
10 – 20 ^e^	3(36.1)	3(36.1)	3(34.7)	2(22.2)	1(11.1)	2(33.6)	3(36.1)	17(45.9)^d^
>20 ^e^	2(22.2)	2(22.2)	3(33.3)	0	2(22.2)	0	0	9(24.3)^d^

*Facility Part of Larger System*								

Yes	0	4(10.8)	7(18.9)	0	0	0	1(2.7)	12(32.4)
No	7(18.9)	2(4.4)	3(8.1)	3(8.1)	4(10.8))	3(8.1)	3(8.1)	25(67.6)

*Preparedness Funding*								

Yes	2(5.4)	2(5.4)	2(5.4)	0	2(5.4)	0	2(5.4)	10(27.0)
No	5(13.5)	4(10.8)	8(21.6)	3(8.1)	2(5.4)	3(8.1)	2(5.4)	27(73.0)

*Accreditation Level*								

Full Standard^b^	5(17.2)	6(20.7)	8(27.6)	1(3.4)	4(13.8)	1(3.4)	4(13.8)	29(78.4)^c^
Requirements for Improvements^b^	0	0	1(33.3)	2(66.7)	0	0	0	3(8.1) ^c^
Not Accredited^b^	2(40.0)	0	1(20.0)	0	0	2(40.0)	0	5(13.5)^c^

Notes

** Urban = Represent hospitals in standard metropolitan statistical area.

* Rural = Represent hospitals in all other statistical areas.

n = Represent the number of respondents in each region.

a = Values in parentheses represent percentages of respondents in each region (total n = 37).

b = Values in parentheses represent percentages of Accreditation in each region.

c = Value in parentheses represent overall percentage in all regions.

d = Value in parentheses represent overall percentage in all regions.

e = Value in parentheses represent percentages in category for each region.

**Table 2:** Frequency distribution of preparedness variables

*Preparedness Variables*	*N =%*			
	*Yes*	*No*	*Yes*	*No*
Documented/Functional preparedness plans	33	4	89.2%	10.8%
Specific preparedness Education/Training	33	4	89.2%	10.8%
Availability of decontamination facilities	28	9	75.7%	24.3%
Ability to increase surge capacity	15	22	40.5%	59.5%
Availability of pharmaceutical procedures/Supplies	21	16	56.8%	43.2%
Laboratory diagnostic capability	3	34	8.1%	91.9%

## References

[1] United States General Accounting Office (2003). “Hospital preparedness: GAO report to the Congressional Committee.”. United States General Accounting Office.

[2] President George Bush “2005 State of the Union Address.”. http://www.CNN.com.

[3] Ashcroft warns of new terrorist threats. http://www.amw.com/features/feature_story.

[4] Martin David “Qaeda still top threat to US.”. http://www.CBSNEWS.com.

[5] Nartker Mike (2005). “Biological attack likely by 2020, Report warns.”. Global Security Newswire.

[6] Linzer Dafna (2005). “Could they pull it off?”. The Washington Post National Weekly Edition.

[7] Tucker Jonathan B (1997). “National Health and Medical Services Response to Incidences of Chemical and Biological Terrorism.”. Journal of the American Medical Association.

[8] Barbara Joseph A, MacIntyre Anthony G, DeAtly Craig A (2002). “Ambulances to nowhere: America’s critical shortfall in medical preparedness for catastrophic terrorism. Journal of Homeland Security.

[9] Anderson Glenn (2003). “Biological and Chemical Terrorism.”. National Conference of State Legislatures Terrorism Preparedness, A Series of Reports About response To Public Health Threats.

[10] United States General Accounting Office (2004). “Hospital preparedness: GAO report to Congressional Committees”. United States General Accounting Office.

[11] The Markle Foundation (2003). “Hospital emergency rooms.”. Council on Foreign Relations.

[12] Henderson Donald A (1999). “The looming threat of bioterrorism,”. Science.

[13] Edgell M, James MJ (1994). “Contaminated casualties: Are we prepared to receive them?”. Journal of Emergency Medicine.

[14] Burgess James L (1999). “Hospital evacuations due to hazardous materials incidents”. American Journal of Emergency Medicine.

[15] United States General Accounting Office (2003). “Hospital preparedness: GAO report to Congressional Committees.”. United States General Accounting Office.

[16] Fraser Michael, Fisher Scott V (2001). “Elements of effective bioterrorism preparedness: A planning primer for local public health agencies.”. National Association of County and City Health Officials.

[17] O’Tool Tara, Mair Michael, Inglesby Thomas V (2002). “Shining light on dark winter.”. Clinical Infectious Disease.

[18] Elliott Victoria S (2003). “Public health’s main fear over bioterrorism: Surge capacity.”. Health & Science.

[19] Ferazani Larry (2004). “Hospitals: Will they be ready when the terrorists are?”. Massachusetts Nurse, Massachusetts Nurses Association.

[20] McGlinchey David “Watchdog group says biological defense remains insufficient.”. http://www.govexec.com/dailyfed/1204/121404d1.htm.

[21] Sprinthall Richard (2000). Basic Statistical Analysis.

[22] Guilford JP (1977). Guidelines for Interpreting the Strength for Values or r: Fundamental Statistics in Psychology and Education.

[23] Fricker Ronald, Jacobson Jerry, Davis Lois (2002). “Measuring and evaluating local preparedness for a chemical or biological terrorist attack.”. RAND Issue Paper.

[24] Code of Federal Regulation, 29, 1910.120.

